# KEYNOTE-434 part B: A phase 1 study evaluating the combination of epacadostat, pembrolizumab, and chemotherapy in Japanese patients with previously untreated advanced non–small-cell lung cancer

**DOI:** 10.1007/s10637-024-01422-6

**Published:** 2024-03-26

**Authors:** Noboru Yamamoto, Miyako Satouchi, Toshihiko Doi, Yutaka Fujiwara, Noriko Yanagitani, Yoshitaka Kawa, Kiyotaka Yoh, Lance Leopold, Mihaela Munteanu, Takashi Sawada, Shirong Han, Kazuo Noguchi, Makoto Nishio

**Affiliations:** 1 Department of Experimental Therapeutics, National Cancer Center Hospital, 5 Chome-1-1 Tsukiji, Tokyo, Japan; 2 Department of Thoracic Oncology, Hyogo Cancer Center, Akashi, Japan; 3 Department of Experimental Therapeutics, National Cancer Center Hospital East, Kashiwa, Japan; 4 Department of Thoracic Oncology, Aichi Cancer Center, Aichi, Japan; 5 Department of Thoracic Medical Oncology, The Cancer Institute Hospital of Japanese Foundation for Cancer Research, Tokyo, Japan; 6 Incyte Corporation, Clinical Development, Wilmington, DE USA; 7 MSD K.K. Oncology Science Unit, Tokyo, Japan

**Keywords:** Indoleamine 2,3-dioxygenase, Non‒small-cell lung cancer, Pembrolizumab, Clinical trial, phase 1

## Abstract

**Background:**

Pembrolizumab plus epacadostat (indoleamine 2,3-dioxygenase-1 inhibitor) was well tolerated in Japanese patients with advanced solid tumors in part A of the nonrandomized, open-label, phase 1 KEYNOTE-434 study (NCT02862457). We report results from part B, which evaluated epacadostat plus pembrolizumab and chemotherapy in Japanese patients with advanced non–small-cell lung cancer (NSCLC).

**Methods:**

Eligible patients aged ≥ 20 years had histologically or cytologically confirmed stage IIIB or IV NSCLC with no prior systemic therapy, and ECOG performance status of 0 or 1. Patients received epacadostat 100 mg orally twice-daily, pembrolizumab 200 mg intravenously every-3-weeks for ≤ 35 cycles, and 4 cycles of chemotherapy (cohort 1: cisplatin plus pemetrexed, non-squamous; cohort 2: carboplatin plus pemetrexed, non-squamous; cohort 3: carboplatin plus paclitaxel, squamous or non-squamous). Primary endpoint was incidence of dose-limiting toxicities (DLTs). Following unfavorable results from other studies, a protocol amendment removed epacadostat from the treatment combination.

**Results:**

Of 19 patients, 7 were enrolled in cohort 1, and 6 each in cohorts 2 and 3. Median follow-up was 13.7 (range, 4.2–27.8) months. Five of 17 (29%) DLT-evaluable patients experienced ≥ 1 DLT (cohort 1, *n* = 1; cohorts 2 and 3, *n* = 2 each); most commonly maculopapular rash (grade 3, *n* = 3) and increased alanine aminotransferase (grade 2, *n* = 1; grade 3, *n* = 2). All patients experienced treatment-related adverse events (AEs); 58% experienced grade 3 or 4 treatment-related AEs. Objective response rate was 47%.

**Conclusion:**

The combination of epacadostat plus pembrolizumab and chemotherapy was found to be tolerable in Japanese patients with advanced NSCLC.

**Trial registration:**

ClinicalTrials.gov, NCT02862457.

## Introduction

In Japan, lung cancer is the leading cause of cancer-related death, and about 50% of cases are diagnosed at stage III or IV [1]. For patients with stage IV non‒small-cell lung cancer (NSCLC), the Japanese Lung Cancer Society recommends that treatment include immune checkpoint inhibitors such as anti‒programmed cell death protein 1 (anti‒PD-1) or anti‒programmed cell death ligand 1 (anti‒PD-L1) antibodies with or without chemotherapy as first-line treatment for NSCLC with PD-L1 tumor proportion score (TPS) ≥ 50% with no *EGFR/ALK* alterations [2]. For a subset of patients (those without a driver oncogene and with PD-L1 TPS < 50% or unknown status), the first-line treatment recommendation is for platinum-containing chemotherapy plus a PD-(L)1 inhibitor [2]. Pembrolizumab, a humanized monoclonal anti‒PD-1 antibody that blocks the interaction of PD-1 with its ligands PD-L1 and PD-L2, is one of the approved therapies [3–6]. Treatment in the first-line setting is associated with 5-year overall survival (OS) rates of 22–32% for pembrolizumab monotherapy in patients with metastatic NSCLC with PD-L1 TPS ≥ 50% [7, 8], and ~ 20% for pembrolizumab plus chemotherapy in patients with non-squamous and squamous metastatic NSCLC [9, 10].

To further improve antitumor activity, combining therapeutic agents that use different mechanisms of action with complementary targets may provide a superior therapeutic effect relative to monotherapies. One such combination was pembrolizumab and epacadostat, an indoleamine 2,3-dioxygenase-1 (IDO1) inhibitor [11, 12]. Epacadostat is an investigational agent that is not approved for the treatment of NSCLC. Preclinical studies showed complementary effects of IDO1 inhibition and checkpoint blockade [13, 14], leading to several clinical trials.

The phase 1/2 ECHO-202/KEYNOTE-037 study demonstrated the safety, tolerability, and antitumor activity of epacadostat plus pembrolizumab in patients with advanced solid tumors, including NSCLC [15]. Of 12 patients with NSCLC, 5 experienced partial response (PR) and 2 experienced stable disease (SD) as the best overall response. Based on these findings, the phase 3 ECHO-305/KEYNOTE-654 [16] and ECHO-306/KEYNOTE-715 [17] studies of epacadostat plus pembrolizumab with or without chemotherapy for metastatic NSCLC were initiated. After epacadostat failed to improve the primary endpoints of progression-free survival (PFS) and overall survival (OS) compared with placebo plus pembrolizumab in a phase 3 study of unresectable or metastatic melanoma [18], the ECHO-305/KEYNOTE-654 [16] and ECHO-306/KEYNOTE-715 [17] studies were amended from phase 3 to phase 2. In the amended KEYNOTE-654 study, the primary endpoint of objective response rate (ORR) was similar in the epacadostat plus pembrolizumab and placebo plus pembrolizumab groups [16]. Similarly, in the amended KEYNOTE-715 study, there was no ORR benefit for epacadostat plus pembrolizumab and chemotherapy versus placebo plus pembrolizumab and chemotherapy [17].

The phase 1 KEYNOTE-434 study was initiated as part of the larger clinical trial program of epacadostat plus pembrolizumab. In KEYNOTE-434, part A (previously published) examined the safety and tolerability of epacadostat alone or in combination with pembrolizumab in Japanese patients with advanced solid tumors [19]. Of 15 patients enrolled, 1 patient receiving epacadostat 100 mg twice daily (BID) and pembrolizumab 200 mg every 3 weeks (Q3W) experienced a dose-limiting toxicity (DLT) of rhabdomyolysis, and grade 3 or 4 treatment-related adverse events (AEs) occurred in 2 patients (13%). Overall, epacadostat plus pembrolizumab was considered well tolerated. The ORR was 27%; 4 patients experienced PR.

We report on part B of KEYNOTE-434, which examined the safety and tolerability of epacadostat plus pembrolizumab and chemotherapy in Japanese patients with advanced or metastatic NSCLC. Results presented here include the available clinical data before a protocol amendment that simplified the trial design and stopped administration of epacadostat in Part B.

## Methods

### Study design and conduct

KEYNOTE-434 (ClinicalTrials.gov, NCT02862457) is a 2-part, open-label, nonrandomized, multicenter, phase 1 study in Japanese patients with advanced solid tumors. Part A was reported previously [19]. Part B was originally designed to evaluate the safety and tolerability of epacadostat plus pembrolizumab and chemotherapy in patients with advanced or metastatic NSCLC.

Patients in part B were enrolled into 1 of 3 cohorts. In all cohorts, patients initially received epacadostat 100 mg orally BID, pembrolizumab 200 mg intravenously Q3W for up to 35 cycles (approximately 2 years), and chemotherapy intravenously for 4 cycles. Chemotherapy comprised cisplatin 75 mg/m^2^ plus pemetrexed 500 mg/m^2^ Q3W in cohort 1 (non-squamous tumors only), carboplatin area under the concentration‒time curve (AUC) 5 mg/mL/min plus pemetrexed 500 mg/m^2^ Q3W in cohort 2 (non-squamous tumors only), and carboplatin AUC 6 mg/mL/min plus paclitaxel 200 mg/m^2^ Q3W in cohort 3 (squamous or non-squamous tumors). However, based on the aforementioned results from the ECHO-305/KEYNOTE-654 and ECHO-306/KEYNOTE-715 studies [16, 17] released on February 19, 2019, the protocol for KEYNOTE-434 was amended. This amendment simplified the trial by removing epacadostat from the treatment combination and removing second-course pembrolizumab except in patients already receiving second-course before the amendment. Additional amendments to the study protocol are described in their respective sections below. Other treatments continued until the maximum number of cycles permitted, radiographic disease progression (PD), unacceptable toxicity, or patient withdrawal. In cohorts 1 and 2, pemetrexed may have continued as maintenance therapy until a discontinuation criterion occurred.

Patients were hospitalized and monitored for DLTs during the first treatment cycle (up to and including study day 21). The study followed a modified toxicity probability interval design [20] that targeted a 30% DLT rate, with an estimated 6 patients enrolled in each cohort for DLT assessment. The treatment combination was considered tolerated if 0 of 3 patients or ≤ 2 of 6 patients in each cohort experienced a DLT. DLTs were defined as any of the following treatment-emergent AEs: grade 4 thrombocytopenia; grade 4 neutropenia lasting > 7 days with appropriate supportive treatment; clinically significant febrile neutropenia; any grade 4 nonhematologic toxicity; any grade 3 laboratory abnormality lasting > 1 week; any other grade 3 nonhematologic toxicity except for nausea or vomiting controlled by medical intervention within 72 h, and rash that resolved to grade 1 before the next scheduled pembrolizumab dose or within 14 days, whichever was longer; grade ≥ 2 episcleritis, uveitis, or iritis; any other toxicity that prevented a patient from receiving 75% of the epacadostat dose or 1 pembrolizumab dose during the DLT evaluation period; or any other toxicity that caused a > 2-week delay in starting cycle 2.

The study was conducted in accordance with all applicable federal and local laws, the International Conference on Harmonization Good Clinical Practice guidelines, and the Declaration of Helsinki. Institutional review boards at each study site approved the study protocol before the study was initiated. All patients provided written informed consent before participating in the study.

### Patient population

Eligible patients were ≥ 20 years old and had histologically or cytologically confirmed stage IIIB or IV NSCLC that was not eligible for anti‒EGFR– or ALK‒directed therapy. Patients were required to have ≥ 1 lesion measurable by computed tomography or magnetic resonance imaging per Response Evaluation Criteria in Solid Tumors (RECIST) version 1.1, an Eastern Cooperative Oncology Group performance status of 0 or 1, life expectancy of ≥ 3 months, and adequate organ function. Patients were eligible if their disease had progressed > 6 months after completing adjuvant therapy for stage I–IIIA disease but should not have received prior systemic therapy for recurrent disease. The provision of tumor tissue for the evaluation of PD-L1 and IDO1 status was optional for patients in part B.

Patients were ineligible for the study if they had previously received immunotherapy (i.e., anti‒PD-[L]1, anti-CD137 or anti‒CTLA-4 agent or IDO1 inhibitor), radiotherapy within 7 days of the first dose of study drug, or radiation therapy to the lung > 30 Gy within 6 months of the first dose of study drug. Other key exclusion criteria were active or previously untreated and clinically unstable central nervous system metastasis and/or carcinomatous meningitis; symptomatic ascites or pleural effusion; active autoimmune disease or infection requiring systemic therapy; receipt of systemic steroid therapy (> 10 mg/day of prednisone equivalent) or immunosuppressive therapy within 1 week of the first dose of study drug; chronic systemic steroids (except for intermittent use of bronchodilators, inhaled steroids, or local steroid injections); history of noninfectious pneumonitis requiring systemic steroids or pneumonitis or interstitial lung disease at the time of enrollment; receipt of a live vaccine within 4 weeks of the first dose of study drug; and inability to interrupt aspirin doses > 1.3 g/day or nonsteroidal anti-inflammatory drugs for a 5-day period (8-day period for long-acting agents; cohorts 1 and 2 only).

### Objectives

The primary endpoint was the incidence of DLTs observed during the DLT evaluation period. AEs were also assessed as part of the safety evaluation. Efficacy was assessed as exploratory endpoints, including investigator-assessed ORR, duration of response (DOR), and PFS per RECIST version 1.1 and OS. Following the protocol amendment, data collection for efficacy endpoints stopped.

### Assessments

AEs were monitored throughout the study and until 90 days after last treatment or 30 days if the patient began new anticancer treatment, whichever came first. Afterwards, serious AEs were only reported if they were related to study treatment. AEs were graded according to the National Cancer Institute Common Toxicity Criteria for Adverse Events version 4.0.

Tumor imaging was performed at screening, then every 6 weeks through week 48, and every 12 weeks thereafter, or more frequently if clinically indicated. Following the protocol amendment, imaging was performed per local standard of care and assessed by the investigator or site radiologist and the use of immune-related RECIST and immune-based RECIST criteria was discontinued. The amendment also removed the follow-up and survival follow-up phases; therefore, the safety follow-up visit constituted the final study visit.

### Statistical analyses

The study planned to enroll 6 patients in each cohort of part B. Safety and efficacy analyses were based on the as-treated population, defined as all patients who received ≥ 1 dose of study drug. Analyses of safety endpoints of DLT and AEs were summarized. For efficacy endpoints, point estimates of ORR and 95% CIs were provided using an exact binomial distribution. DOR, PFS, and OS were tabulated across dose levels and summarized descriptively using the Kaplan-Meier method.

## Results

### Study population

Between February 22, 2018 and February 21, 2019, 19 patients were enrolled at 4 sites in Japan and were included in the safety and efficacy analyses in part B of the study. In cohort 1 (*n* = 7), patients with non-squamous tumors received cisplatin plus pemetrexed; in cohort 2 (*n* = 6), patients with non-squamous tumors received carboplatin plus pemetrexed, and in cohort 3 (*n* = 6), patients with squamous or non-squamous tumors received carboplatin plus paclitaxel; all patients were included in the safety and efficacy analyses. All patients received pembrolizumab and epacadostat, plus the indicated chemotherapy, excluding 1 patient in cohort 3 who did not receive epacadostat. Based on the aforementioned results from the ECHO-305/KEYNOTE-654 and ECHO 306/KEYNOTE-715 studies, 9 patients discontinued epacadostat (cohort 1, *n* = 3; cohort 2, *n* = 3; and cohort 3, *n* = 3).

At the database cutoff date of March 17, 2021, 4 patients had completed treatment (cohort 1, *n* = 2; cohort 2, *n* = 0; cohort 3, *n* = 2) and the remaining 15 had discontinued treatment due to PD (cohort 1, *n* = 3; cohort 2, *n* = 3; cohort 3, *n* = 2), AEs (*n* = 0, *n* = 2, and *n* = 2), patient withdrawal (*n* = 1, *n* = 1, and *n* = 0), and physician decision (*n* = 1, *n* = 0, and *n* = 0). Most patients were male (74%) and had not received prior neoadjuvant or adjuvant therapy (89%) (Table 1).

**Table 1:** Patient demographics and baseline disease characteristics

	Cohort 1^a^ *n* = 7	Cohort 2^b^ *n* = 6	Cohort 3^c^ *n* = 6	Total *N* = 19
Sex				
Male	6 (86)	3 (50)	5 (83)	14 (74)
Female	1 (14)	3 (50)	1 (17)	5 (26)
Age, median (range), y	58.0 (45‒67)	69.0 (63‒73)	66.0 (44‒74)	64.0 (44‒74)
ECOG PS				
0	5 (71)	2 (33)	2 (33)	9 (47)
1	2 (29)	4 (67)	4 (67)	10 (53)
Prior adjuvant therapy				
Yes	0	1 (17)	1 (17)	2 (11)
No	7 (100)	5 (83)	5 (83)	17 (89)

All data are n (%) unless otherwise specified

AUC, area under the concentration‒time curve; BID, twice daily; ECOG PS, Eastern Cooperative Oncology Group performance status; NSCLC, non‒small-cell lung cancer; Q3W, every 3 weeks

^a^Epacadostat 100 mg BID plus pembrolizumab 200 mg Q3W in combination with cisplatin 75 mg/m^2^ and pemetrexed 500 mg/m^2^ Q3W in patients with non-squamous NSCLC

^b^Epacadostat 100 mg BID plus pembrolizumab 200 mg Q3W in combination with carboplatin AUC 5 and pemetrexed 500 mg/m^2^ Q3W in patients with non-squamous NSCLC

^c^Epacadostat 100 mg BID plus pembrolizumab 200 mg Q3W in combination with carboplatin AUC 6 and paclitaxel 200 mg/m^2^ Q3W in patients with squamous and non-squamous NSCLC

The median (range) duration of time from first dose to end of trial (or minimum of the death date or database cutoff date, if end-of-trial date was missing) was 13.7 months (4.2‒27.8 months) in the overall population, 17.2 (10.1‒26.0) months in cohort 1, 8.2 (4.2‒16.8) months in cohort 2, and 16.0 (5.6‒27.8) months in cohort 3. Median (range) duration of exposure to study drug, defined as the time between the dates of the first and last doses, was 5.7 (0.3‒26.6) months in the overall population, 9.4 (0.4‒25.1) months in cohort 1, 1.0 (0.3‒7.8) months in cohort 2, and 4.9 (0.3‒26.6) months in cohort 3.

### Safety

Overall, 5 of the 17 DLT-evaluable patients (29%) experienced DLTs: 1 patient in cohort 1 and 2 patients each in cohorts 2 and 3. The most common DLTs were maculopapular rash (grade 3, *n* = 3) and increased alanine aminotransferase (grade 2, *n* = 1; grade 3, *n* = 2) (Table 2). The median time to onset of DLTs was 6 to 12 days from the start of study treatment. In cohort 1, 7 patients were allocated, but 1 patient was excluded from the DLT-evaluable population due to the use of prohibited concomitant medication. One of the 6 patients (17%) experienced a DLT of grade 3 maculopapular rash. In cohort 2, 2 of the 6 patients (33%) experienced DLTs (grade 3 increased alanine aminotransferase in 1 patient and grade 3 maculopapular rash in 2 patients). In cohort 3, 6 patients were allocated, but 1 patient was excluded from the DLT-evaluable population due to failure to receive at least 1 dose of epacadostat. The patient was not replaced with a new one due to the protocol amendment to remove epacadostat from the treatment combination; therefore, cohort 3 included 5 DLT-evaluable patients and did not reach the target number of 6 patients for DLT assessment. Two of the 5 patients (40%) experienced DLTs; 1 patient had grade 3 increased alanine aminotransferase, and the other patient had grade 2 events of nausea, increased alanine aminotransferase, and increased aspartate aminotransferase, and grade 1 vomiting and pyrexia. These toxicities in cohort 3 were manageable with intervention and/or suspension of treatment but considered DLTs because both patients were unable to receive 75% of epacadostat during the DLT observation period.

**Table 2:** Summary of DLTs

	Cohort 1^a^ *n* = 6	Cohort 2^b^ *n* = 6	Cohort 3^c^ *n* = 5	Total *N* = 17
Patients with DLT, n (%)	1 (17)	2 (33)	2 (40)	5 (29)
Time to onset, median (range), days,	12 (NA)	10 (5‒13)	6 (3‒19)	9 (3‒19)
DLTs, n				
Grade 3 maculopapular rash	1	2	0	3
Grade 3 increased alanine aminotransferase	0	1	1^d^	2
Grade 2 increased alanine aminotransferase^d^	0	0	1	1
Grade 2 increased aspartate aminotransferase^d^	0	0	1	1
Grade 2 nausea^d^	0	0	1	1
Grade 1 pyrexia^d^	0	0	1	1
Grade 1 vomiting^d^	0	0	1	1

AUC, area under the concentration‒time curve; BID, twice daily; DLT, dose-limiting toxicity; NA, not applicable; NSCLC, non‒small-cell lung cancer; Q3W, every 3 weeks

^a^Epacadostat 100 mg BID plus pembrolizumab 200 mg Q3W in combination with cisplatin 75 mg/m^2^ and pemetrexed 500 mg/m^2^ Q3W in patients with non-squamous NSCLC

^b^Epacadostat 100 mg BID plus pembrolizumab 200 mg Q3W in combination with carboplatin AUC 5 and pemetrexed 500 mg/m^2^ Q3W in patients with non-squamous NSCLC

^c^Epacadostat 100 mg BID plus pembrolizumab 200 mg Q3W in combination with carboplatin AUC 6 and paclitaxel 200 mg/m^2^ Q3W in patients with squamous and non-squamous NSCLC

^d^Event was considered a DLT because the patient was unable to receive 75% of epacadostat dose during the DLT observation period

Among the 5 patients who experienced DLTs, 3 patients (56-year-old male, 70-year-old female, and 68-year-old female) in cohorts 1 and 2 experienced grade 3 maculopapular rash, and 2 patients (70-year-old female and 71-year-old female) in cohorts 2 and 3 experienced grade 3 increased alanine aminotransferase. Each of these was considered a DLT by the primary investigator and the sponsor given the safety profile of epacadostat and led to study treatment discontinuation. Median (range) onset of maculopapular rash was 12 (10–13) days and of increased alanine aminotransferase was after 6.5 (5–8) days, all following the first and only dose of pembrolizumab and during treatment with epacadostat. All patients received treatment for the DLT (steroid therapy, etc.). All incidences of maculopapular rash resolved in a median of 35 (range, 33–37) days; whereas 1 patient recovered from increased alanine aminotransferase in 81 days, and the other was lost to follow-up.

All patients experienced treatment-related AEs and 11 (58%) experienced grade 3 or 4 treatment-related AEs; there were no treatment-related deaths (Table 3). The most common grade 3 or 4 treatment-related AEs were decreased neutrophil count, maculopapular rash, and decreased white blood cell count (*n* = 3 each; Table 3). Treatment-related AEs led to discontinuation of study treatment in 5 patients (26%): none in cohort 1; 2 patients in cohort 2 due to increased alanine aminotransferase, increased aspartate aminotransferase, and maculopapular rash; and 3 patients in cohort 3 due to anaphylactic reaction, increased alanine aminotransferase, and dizziness.

**Table 3:** Summary of AEs

AE, n (%)	Cohort 1^a^ *n* = 7	Cohort 2^b^ *n* = 6	Cohort 3^c^ *n* = 6	Total *N* = 19
Any AE	7 (100)	6 (100)	6 (100)	19 (100)
Treatment-related AE	7 (100)	6 (100)	6 (100)	19 (100)
Grade 3/4	2 (29)	5 (83)	4 (67)	11 (58)
Led to death	0	0	0	0
Serious	1 (14)	1 (17)	2 (33)	4 (21)
Led to treatment discontinuation	0	2 (33)	3 (50)	5 (26)
Treatment-related AEs occurring in ≥ 2 patients in any cohort				
Nausea	4 (57)	5 (83)	2 (33)	11 (58)
Increased alanine aminotransferase	4 (57)	2 (33)	3 (50)	9 (47)
Constipation	4 (57)	3 (50)	1 (17)	8 (42)
Increased aspartate aminotransferase	3 (43)	2 (33)	3 (50)	8 (42)
Decreased appetite	2 (29)	3 (50)	2 (33)	7 (37)
Pyrexia	3 (43)	1 (17)	2 (33)	6 (32)
Maculopapular rash	2 (29)	3 (50)	0	5 (26)
Malaise	2 (29)	1 (17)	2 (33)	5 (26)
Alopecia	0	0	4 (67)	4 (21)
Anemia	1 (14)	2 (33)	1 (17)	4 (21)
Decreased neutrophil count	1 (14)	2 (33)	1 (17)	4 (21)
Decreased white blood cell count	1 (14)	1 (17)	2 (33)	4 (21)
Arthralgia	0	0	3 (50)	3 (16)
Decreased platelet count	0	2 (33)	1 (17)	3 (16)
Dysgeusia	1 (14)	2 (33)	0	3 (16)
Peripheral sensory neuropathy	1 (14)	0	2 (33)	3 (16)
Stomatitis	3 (43)	0	0	3 (16)
Edema	2 (29)	0	0	2 (11)
Hypothyroidism	0	0	2 (33)	2 (11)
Myalgia	0	0	2 (33)	2 (11)
Neutropenia	0	0	2 (33)	2 (11)

AE, adverse event; AUC, area under the concentration‒time curve; BID, twice daily; NSCLC, non‒small-cell lung cancer; Q3W, every 3 weeks

^a^Epacadostat 100 mg BID plus pembrolizumab 200 mg Q3W in combination with cisplatin 75 mg/m^2^ and pemetrexed 500 mg/m^2^ Q3W in patients with non-squamous NSCLC

^b^Epacadostat 100 mg BID plus pembrolizumab 200 mg Q3W in combination with carboplatin AUC 5 and pemetrexed 500 mg/m^2^ Q3W in patients with non-squamous NSCLC

^c^Epacadostat 100 mg BID plus pembrolizumab 200 mg Q3W in combination with carboplatin AUC 6 and paclitaxel 200 mg/m^2^ Q3W in patients with squamous and non-squamous NSCLC

Treatment-related AEs led to interruption of study treatment in 9 patients (47%). In cohort 1, treatment interruption occurred in 5 patients due to treatment-related AEs of pyrexia (*n* = 2) and vertigo, edema, decreased neutrophil count, dizziness, pneumonitis, and maculopapular rash (*n* = 1 each). In cohort 2, treatment-related AEs interrupted treatment in 2 patients due to pyrexia, decreased neutrophil count, and maculopapular rash (*n* = 1 each). In cohort 3, treatment-related AEs interrupted treatment in 2 patients due to increased alanine aminotransferase and increased aspartate aminotransferase (*n* = 2 each) and nausea, vomiting, and pyrexia (*n* = 1 each).

### Efficacy

**Fig. 1:**
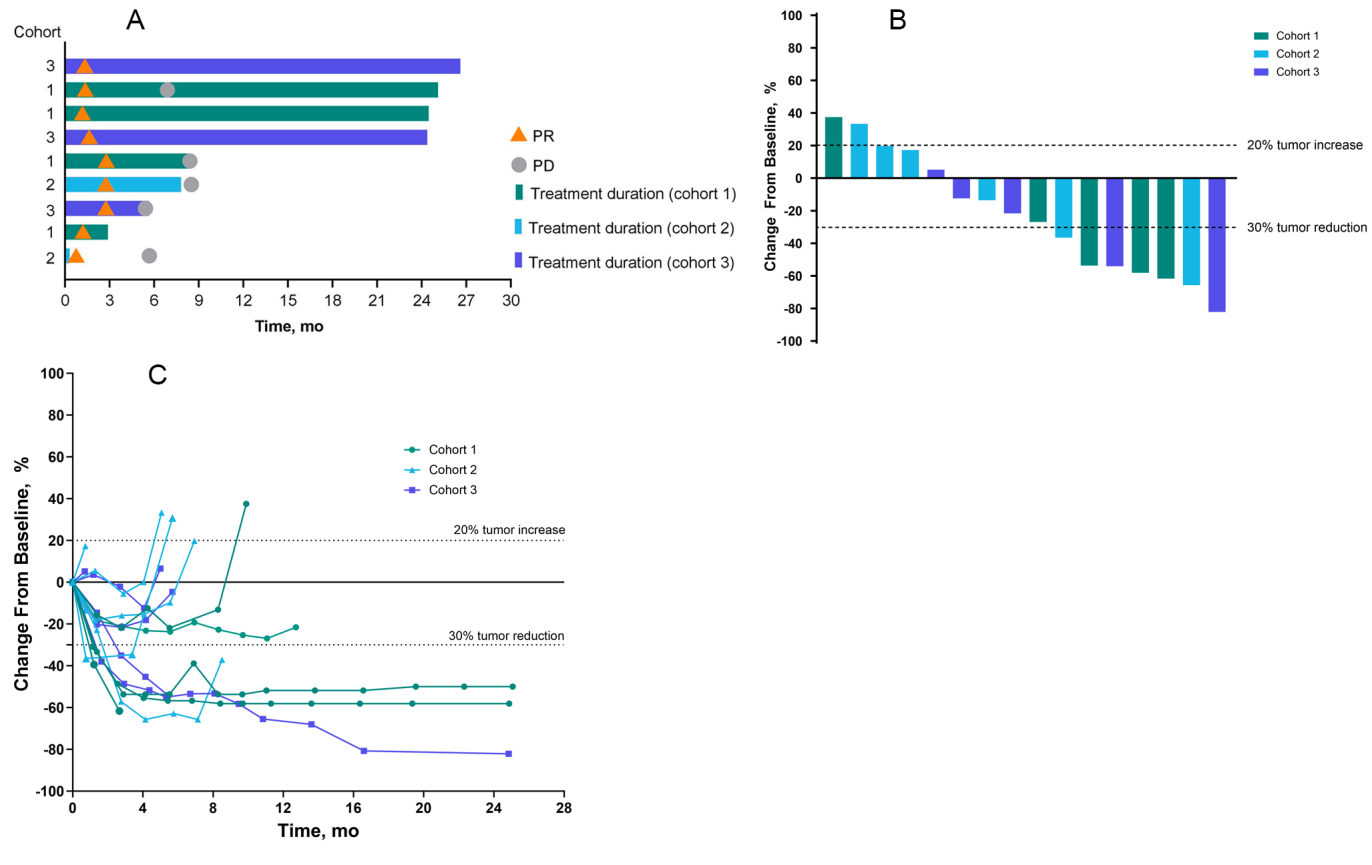
(**A**) Treatment duration and time to response per RECIST version 1.1 by investigator review. (**B**) Maximum percent change from baseline in target tumor lesions for individual patients per RECIST version 1.1 by investigator review. (**C**) Percentage change from baseline in tumor lesion size over time for individual patients. All cohorts received epacadostat 100 mg twice daily plus pembrolizumab 200 mg every 3 weeks (Q3W). Additionally, patients in cohort 1 (non-squamous NSCLC) received cisplatin 75 mg/m^2^ and pemetrexed 500 mg/m^2^, cohort 2 (non-squamous NSCLC) received carboplatin area under the concentration–time curve (AUC) 5 mg/mL/min and pemetrexed 500 mg/m^2^, and cohort 3 (squamous and non-squamous NSCLC) received carboplatin AUC 6 mg/mL/min and paclitaxel 200 mg/m^2^. PD, progressive disease; PR, partial response; RECIST, Response Evaluation Criteria in Solid Tumors

Overall, 9 patients (47%) had an objective response, all of which were PRs (Fig. 1A). The ORR (95% CI) was 57% (18‒90%) in cohort 1, 33% (4‒78%) in cohort 2, and 50% (12‒88%) in cohort 3 (Table 4). The median (range) time to response was 1.4 (0.8‒2.8) months in the overall population, 1.3 (1.2‒2.8) months in cohort 1, 1.8 (0.8‒2.8) months in cohort 2, and 1.6 (1.3‒2.8) months in cohort 3. Median (range) duration of response was 5.7 (1.5 + to 26.4+) months in the overall population, 5.7 (1.5 + to 23.7+) months in cohort 1, 5.4 (5.0 to 5.8) months in cohort 2, and not reached (NR; 2.7 to 26.4 + months) in cohort 3. No patient had ongoing response at the time of data cutoff. Among the 9 patients who discontinued epacadostat following the protocol amendment, 5 had PR (cohort 1, *n* = 1; cohort 2, *n* = 1; cohort 3, *n* = 3) and 4 had SD (cohort 1, *n* = 2; cohort 2, *n* = 2). All 9 patients received at least 3 months of epacadostat, with the exception of 1 patient with SD who received < 2 months of epacadostat.

**Table 4:** Best Overall Response per RECIST Version 1.1 by Investigator Assessment

	Cohort 1^a^ *n* = 7	Cohort 2^b^ *n* = 6	Cohort 3^c^ *n* = 6	Total *N* = 19
ORR (95% CI), %	57 (18‒90)	33 (4‒78)	50 (12‒88)	47 (24‒71)
Best overall response, n (%)				
CR	0	0	0	0
PR	4 (57)	2 (33)	3 (50)	9 (47)
SD	2 (29)	2 (33)	2 (33)	6 (32)
Progressive disease	0	1 (17)	0	1 (5)
Not evaluable	0	1 (17)	1 (17)	2 (11)
No assessment	1 (14)	0	0	1 (5)
Disease control rate (CR + PR + SD), n (%)	6 (86)	4 (67)	5 (83)	15 (79)
Time to response, median (range), months	1.3 (1.2‒2.8)	1.8 (0.8‒2.8)	1.6 (1.3‒2.8)	1.4 (0.8‒2.8)
Duration of response, median (range), months	5.7 (1.5 + to 23.7+)	5.4 (5.0 to 5.8)	Not reached (2.7 to 26.4+)	5.7 (1.5 + to 26.4+)

AUC, area under the concentration‒time curve; BID, twice daily; CR, complete response; NSCLC, non‒small-cell lung cancer; ORR, objective response rate; PR, partial response; Q3W, every 3 weeks; SD, stable disease

‘+’ indicates there was no disease progression at the time of last disease assessment

^a^Epacadostat 100 mg BID plus pembrolizumab 200 mg Q3W in combination with cisplatin 75 mg/m^2^ and pemetrexed 500 mg/m^2^ Q3W in patients with non-squamous NSCLC

^b^Epacadostat 100 mg BID plus pembrolizumab 200 mg Q3W in combination with carboplatin AUC 5 and pemetrexed 500 mg/m^2^ Q3W in patients with non-squamous NSCLC

^c^Epacadostat 100 mg BID plus pembrolizumab 200 mg Q3W in combination with carboplatin AUC 6 and paclitaxel 200 mg/m^2^ Q3W in patients with squamous and non-squamous NSCLC

Among 16 patients evaluable for change from baseline in target lesions, 11 had a maximum change associated with tumor reduction (Fig. 1B). This included 7 patients with greater than 30% reduction in tumor size from baseline. There were 5 patients whose maximum change from baseline was tumor growth (Fig. 1B); some of these patients initially experienced tumor reduction, but tumor growth occurred at a later time point (Fig. 1C).

At data cutoff, events of PD or death had occurred in 14 patients (74%; cohort 1, *n* = 4; cohort 2, *n* = 6; cohort 3, *n* = 4). The median (95% CI) PFS was 7.7 (5.4‒11.1) months in the overall population, 9.9 (6.9–not estimable [NE]) months in cohort 1, 6.3 (0.7–NE) months in cohort 2, and 8.6 (5.0–NE) months in cohort 3. The 12-month PFS rates were 18% in the overall population, 20% in cohort 1, NR in cohort 2, and 33% in cohort 3.

Overall, 3 patients (16%) had died at the time of data cutoff, including 2 patients in cohort 2 and 1 in cohort 3. The median OS was NR (95% CI, 11.6 months‒NE) in the overall population or in any of the cohorts. The 12-month OS rate was 80% in the overall population, 100% in cohort 1, 63% in cohort 2, and 75% in cohort 3.

## Discussion

In part B of the KEYNOTE-434 study, the combination of epacadostat plus pembrolizumab and chemotherapy was found to be tolerable in Japanese patients with previously untreated advanced or metastatic NSCLC in 6 patients in cohorts 1 (cisplatin plus pemetrexed) and 2 (carboplatin plus pemetrexed), and 5 patients in cohort 3 (carboplatin plus paclitaxel). Because the study protocol was amended to discontinue epacadostat at the time, cohort 3 did not reach the targeted number of patients (*n* = 6), and the DLT assessment was based on 5 patients only. Among 5 patients (29%) who experienced DLTs across all cohorts, maculopapular rash and increased alanine aminotransferase were the most common. Though this study was limited by the small number of patients and the short follow-up due to the protocol amendment, antitumor activity was observed across all cohorts, with nearly half of the patients achieving an objective response. Most patients were alive at the time of data cutoff.

Similar to part A of the study (epacadostat alone or in combination with pembrolizumab in Japanese patients with solid tumors), all patients in part B experienced treatment-related AEs, and no patient experienced a fatal treatment-related AE. However, there was some variation in the incidence and types of AEs experienced between parts A and B. Common treatment-related AEs in part A included rash (27%), diarrhea (27%), pruritus (13%), and increased blood thyroid‒stimulating hormone (13%). The addition of chemotherapy to the treatment regimen in part B likely influenced the AE profile relative to part A, as reflected by the most frequent AEs, which were nausea, increased alanine aminotransferase, increased aspartate aminotransferase, and constipation in part B. This may also account for the greater number of DLTs observed in part B compared to part A, in which only 1 of 15 patients with advanced solid tumors experienced a DLT of rhabdomyolysis with epacadostat plus pembrolizumab [19]. Altogether, the safety profile in part B is consistent with those associated with each treatment component and as reported previously, including in the ECHO-305/KEYNOTE-654 and ECHO-306/KEYNOTE-715 studies of epacadostat plus pembrolizumab with and without chemotherapy in patients with NSCLC [5, 16, 17, 21, 22]. The AE profile is also similar to studies of epacadostat in combination with other immune checkpoint inhibitors in patients with NSCLC, where the most common treatment-related AEs included fatigue, nausea, and decreased appetite [23, 24].

Antitumor activity was observed regardless of tumor histology. The ORR in part B (47%) was slightly higher than that observed with epacadostat plus pembrolizumab in part A of the study (27%) and in ECHO-305/KEYNOTE-654 (33%) [16] and with epacadostat and pembrolizumab plus chemotherapy in ECHO-306/KEYNOTE-715 (26%) [17]. Although cross-study comparisons should be made with caution, ORR was higher in Japanese patients enrolled in KEYNOTE-189 and KEYNOTE-407 studies, which examined pembrolizumab plus chemotherapy in patients with previously untreated metastatic non-squamous and squamous NSCLC, respectively, and ranged from 56% to 68% [22, 25].

While most patients were alive at the time of data cutoff, follow-up was limited because epacadostat treatment and efficacy follow-up were stopped early as of the protocol amendment. While the impact of discontinuing epacadostat on ORR and PFS cannot be completely discounted, given the median time to tumor response (among all patients) was 1.4 months (range, 0.8–2.8), and all but 1 of the 9 patients who discontinued epacadostat following protocol amendment received ≥ 3 months of treatment, the impact on these outcomes was likely limited. Interpretation of the current study results is also limited by the lack of a comparator group, small sample size, and protocol amendments to remove epacadostat from the treatment regimen.

In conclusion, the combination of epacadostat plus pembrolizumab and chemotherapy was generally tolerated and demonstrated antitumor activity in Japanese patients with advanced or metastatic NSCLC. These results are consistent with prior reports of epacadostat plus pembrolizumab with or without chemotherapy in patients with NSCLC [15–17, 19], although the study protocol was amended and epacadostat was discontinued early. Based on the evidence to date, the combination of epacadostat plus pembrolizumab and chemotherapy may not provide added benefit compared with pembrolizumab with or without chemotherapy, and the development of this combination has therefore been discontinued. Pembrolizumab with and without chemotherapy remains a standard of care in patients with advanced or metastatic NSCLC.

## Data Availability

Merck Sharp & Dohme LLC, a subsidiary of Merck & Co., Inc., Rahway, NJ, USA (MSD) is committed to providing qualified scientific researchers access to anonymized data and clinical study reports from the company’s clinical trials for the purpose of conducting legitimate scientific research. MSD is also obligated to protect the rights and privacy of trial participants and, as such, has a procedure in place for evaluating and fulfilling requests for sharing company clinical trial data with qualified external scientific researchers. The MSD data sharing website (available at: http://engagezone.msd.com/ds_documentation.php) outlines the process and requirements for submitting a data request. Applications will be promptly assessed for completeness and policy compliance. Feasible requests will be reviewed by a committee of MSD subject matter experts to assess the scientific validity of the request and the qualifications of the requestors. In line with data privacy legislation, submitters of approved requests must enter into a standard data-sharing agreement with MSD before data access is granted. Data will be made available for request after product approval in the US and EU or after product development is discontinued. There are circumstances that may prevent MSD from sharing requested data, including country or region-specific regulations. If the request is declined, it will be communicated to the investigator. Access to genetic or exploratory biomarker data requires a detailed, hypothesis-driven statistical analysis plan that is collaboratively developed by the requestor and MSD subject matter experts; after approval of the statistical analysis plan and execution of a data-sharing agreement, MSD will either perform the proposed analyses and share the results with the requestor or will construct biomarker covariates and add them to a file with clinical data that is uploaded to an analysis portal so that the requestor can perform the proposed analyses.
